# A new method to compile global multi-hazard event sets

**DOI:** 10.1038/s41598-023-40400-5

**Published:** 2023-08-23

**Authors:** Judith N. Claassen, Philip J. Ward, James Daniell, Elco E. Koks, Timothy Tiggeloven, Marleen C. de Ruiter

**Affiliations:** 1 Institute for Environmental Studies, Vrije Universiteit Amsterdam, Amsterdam, The Netherlands; 2 Deltares, Delft, The Netherlands; 3 Risklayer GmbH, Karlsruhe, Germany; 4 CEDIM, Karlsruhe Institute of Technology, Karlsruhe, Germany

**Keywords:** Climate sciences, Hydrology, Natural hazards

## Abstract

This study presents a new method, the MYRIAD-Hazard Event Sets Algorithm (MYRIAD-HESA), that compiles historically-based multi-hazard event sets. MYRIAD-HESA is a fully open-access method that can create multi-hazard event sets from any hazard events that occur on varying time, space, and intensity scales. In the past, multi-hazards have predominately been studied on a local or continental scale, or have been limited to specific hazard combinations, such as the combination between droughts and heatwaves. Therefore, we exemplify our approach by compiling a global multi-hazard event set database, spanning from 2004 to 2017, which includes eleven hazards from varying hazard classes (e.g. meteorological, geophysical, hydrological and climatological). This global database provides new scientific insights on the frequency of different multi-hazard events and their hotspots. Additionally, we explicitly incorporate a temporal dimension in MYRIAD-HESA, the time-lag. The time-lag, or time between the occurrence of hazards, is used to determine potentially impactful events that occurred in close succession. Varying time-lags have been tested in MYRIAD-HESA, and are analysed using North America as a case study. Alongside the MYRIAD-HESA, the multi-hazard event sets, MYRIAD-HES, is openly available to further increase the understanding of multi-hazard events in the disaster risk community. The open-source nature of MYRIAD-HESA provides flexibility to conduct multi-risk assessments by, for example, incorporating higher resolution data for an area of interest.

## Introduction

On August 14th 2021, Haiti was hit by a magnitude 7.2 earthquake that destroyed significant parts of its infrastructure. It claimed approximately 2000 lives, and injured over 12,000 people^1^. Three days later, tropical storm Grace raged over the country, forcing the population to shelter in damaged buildings that had become unstable due to the destructive effect of the earthquake. Moreover, Grace produced heavy rainfall over the affected area causing several landslides, blocking roads, and hampering rescue efforts as well as humanitarian aid missions^1^. Additionally, Haiti was in the midst of the COVID-19 pandemic, which further hindered disaster response because of the stringent health and safety protocols^2^. These hazardous events that hit Haiti in close succession can be classified as a multi-hazard event. Multi-hazard is defined by the United Nations Office for Disaster Risk Reduction^3^ as “*The specific contexts where hazardous events may occur simultaneously, cascadingly or cumulatively over time, and taking into account the potential interrelated effects.*”

The risks generated by such a multi-hazard event are referred to as multi-risk^4^. The necessity for a better understanding of multi-risk is recognized internationally in the IPCC 6th Assessment Report and the Sendai Framework for Disaster Risk Reduction^5,6^. To increase this understanding, the multi-hazard component (i.e., all the individual hazards a location faces and their interaction) needs to be better determined and understood^7–12^.

In the field of multi-hazards, various multi-hazard interrelations have been defined. We divide these interrelations into four categories, noting that they are not mutually exclusive:

1. Compound hazards: Compound weather and climate events are defined as a combination of multiple drivers and/or hazards that contribute to risk^13^
2. Consecutive hazards: Two or more disasters that occur in succession, and whose direct impacts overlap spatially before recovery from a previous event is completed^10^
3. Triggering hazards: One hazard causes another hazard to occur, which can result in hazard chains, networks, or cascades^14^.
4. Amplifying hazards: When one hazard increases the probability of another hazard occurring^14^.

These complex multi-hazard interrelations have been studied in the past. However, these studies predominately focus on a local or continental scale, and have been limited to specific hazard combinations (pairs), such as the joint occurrence of wind and precipitation^15–25^. Research that has analysed a more extensive array of hazard interactions on a global scale pays particular attention to compound hazard events, which are confined to the interaction of climate hazards^11,23,26,27^. While the extensive compound event research has contributed significantly to our understanding of multivariate hazards, it is also important to consider hazards from various hazard classes (i.e., geophysical, meteorological, climatological, and hydrological), as the events in Haiti in 2021 have shown^10^.

Furthermore, the Haiti example highlights that the time after a first natural hazard can be of importance, where the initial hazard, the earthquake, left the country more vulnerable and exposed to a tropical cyclone three days later^10,28^. Despite the awareness that time between hazards is important to consider, there are only a few studies that assess the temporal aspect across hazard classes, possibly due to its complexity^29–31^. Hence, more work is needed to understand the temporal aspect of multi-hazards events while accounting also for all natural hazard classes and including more than two hazards at the time.

Whilst the aforementioned studies have made advances in identifying links between different hazards, the lack of a method to compile a coherent database on multi-hazard events has been highlighted in the literature as a major challenge in advancing our understanding of multi-hazard risk^7^. Therefore, in this study we present a state-of-the-art method for compiling global multi-hazard event sets, the MYRIAD—Hazard Event Sets Algorithm (MYRIAD-HESA), and apply the method to compile the first global multi-hazard event set database (MYRIAD-HES). The aim of this paper is to use the database to demonstrate how frequently multi-hazards of varying combinations occur, and where their hotspots are located, while accounting for different time-lags in between hazards. The MYRIAD-HES incorporates historic single-hazard events data (including geophysical, meteorological, hydrological, and climatological hazards) collected from various sources with an overlapping timespan from 2004 to 2017. The MYRIAD-HESA has been developed to compile multi-hazard event sets based on spatial and temporal overlap. The algorithm enables a time-lag to be introduced for each hazard, where multiple hazards are considered a multi-hazard event if they occur within a given time-lag. To consider time in between events, various time-lags are tested to understand the effect that a time-lag has on the number and type of multi-hazard events.

## Methodology

This methodology first provides an explanation of how the event sets have been developed with and without a time-lag in section “Compiling multi-hazard event sets”. Following this, the single-hazard input data and data processing methods are described in section “Single natural hazard datasets”.

### Compiling multi-hazard event sets

**Figure 1:**
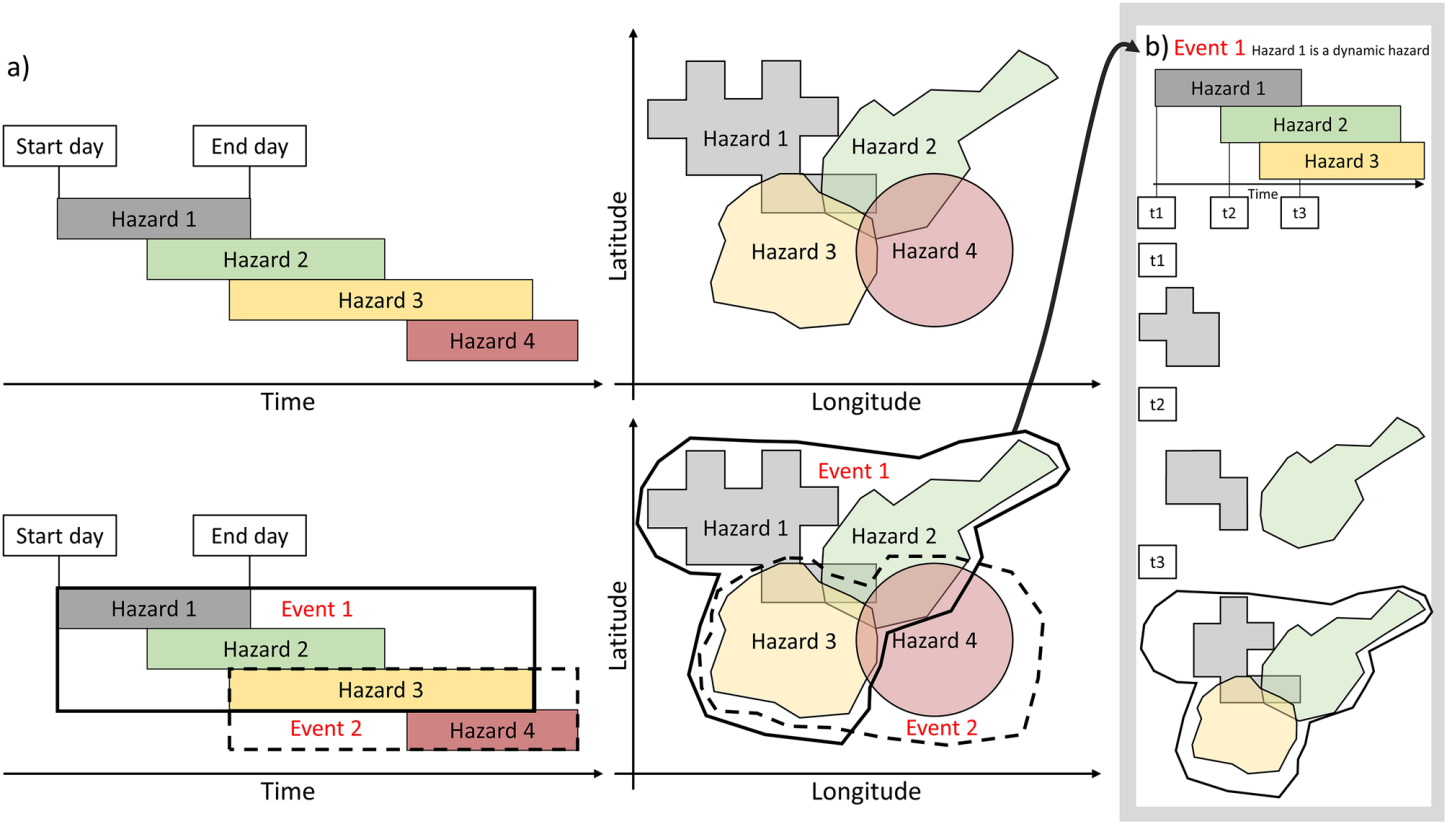
Example of how MYRIAD-HESA operates without a time-lag. This figure shows both hazard pairs and hazard groups. (**a**) Hazards are a hazard group if all hazards overlap with each other in space and time as a pair. Here, there are two hazard groups, which are referred to as Events. Event 1 is encompassed by the black solid line, while Event 2 is encompassed by the black dashed line. Event 1 consists of three hazard pairs between Hazard 1, 2, and 3. Event 2 consists of one hazard pair between Hazard 3 and 4. (**b**) A dynamic hazard has to overlap with the other hazards during at least one of the overlapping time-steps. Here, Hazard 1 is a dynamic hazard. Therefore, it’s event polygon can change over time. Hazard 2 and Hazard 3 are not dynamic hazards. Their polygons remain the same between their start time and end time.

In this section, we describe MYRIAD-HESA, the algorithm used to compile MYRIAD-HES, of which the code is publicly available^32^. A visual overview is presented in Fig. 1, and the steps are further explained below.

To produce the multi-hazard event sets, we require information on where and when a hazard occurred by using single hazard event data. Each single hazard footprint consists of a polygon that represents the spatial extent where the hazard occurred (hereinafter referred to as event polygon). Additionally, each hazard has a starting date, an end date, and a measured intensity if applicable.

In this study, two hazards are a pair if both their event polygon and timeframe overlap, as seen in Fig. 1a. For example, Hazard 2 and Hazard 4 overlap in space, but not in time, and are therefore not a pair. However, Hazard 4 does overlap in space and time with Hazard 3, these are a pair. A hazard can also be classified as a dynamic hazard. Dynamic hazards are those for which there is information on their spatial development through time. For example, a wildfire can spread or diminish over the span of its lifetime, leading to multiple event polygons for each individual timestep. Therefore, if one or both hazards in a hazard pair are dynamic hazards, the dynamic polygons at each time step have to be checked to see whether the two hazards truly overlap at one point in space and time before they can be considered a pair (as seen in Fig. 1b). Each individual hazard is noted by a unique id. A hazard pair is therefore noted as a row with two columns where the first column includes the id of the first hazard and the second column includes the id of the second hazard. The pairs are all ordered based on the start date of the individual hazards. However, hazards in a group can also have the same start date. In this case, the hazard that occurs first in the data will be listed as the first hazards. Hence, no prior hazard relationship knowledge is implemented into the method.

After all the hazard pairs are known, they can be put into hazard groups. These hazard groups are the final multi-hazard events. A pair can form a group with other hazard pairs if all individual hazards overlap with one another. For example, if there is a hazard pair (Hazard 1_,_ Hazard 2) and a hazard pair (Hazard 2_,_ Hazard 3), these two pairs can form a group if hazard pair (Hazard 1_,_ Hazard 3) also exists. In this case the hazard event will consist of Hazard 1_,_ Hazard 2 and Hazard 3 (see Fig. 1a).

**Figure 2:**
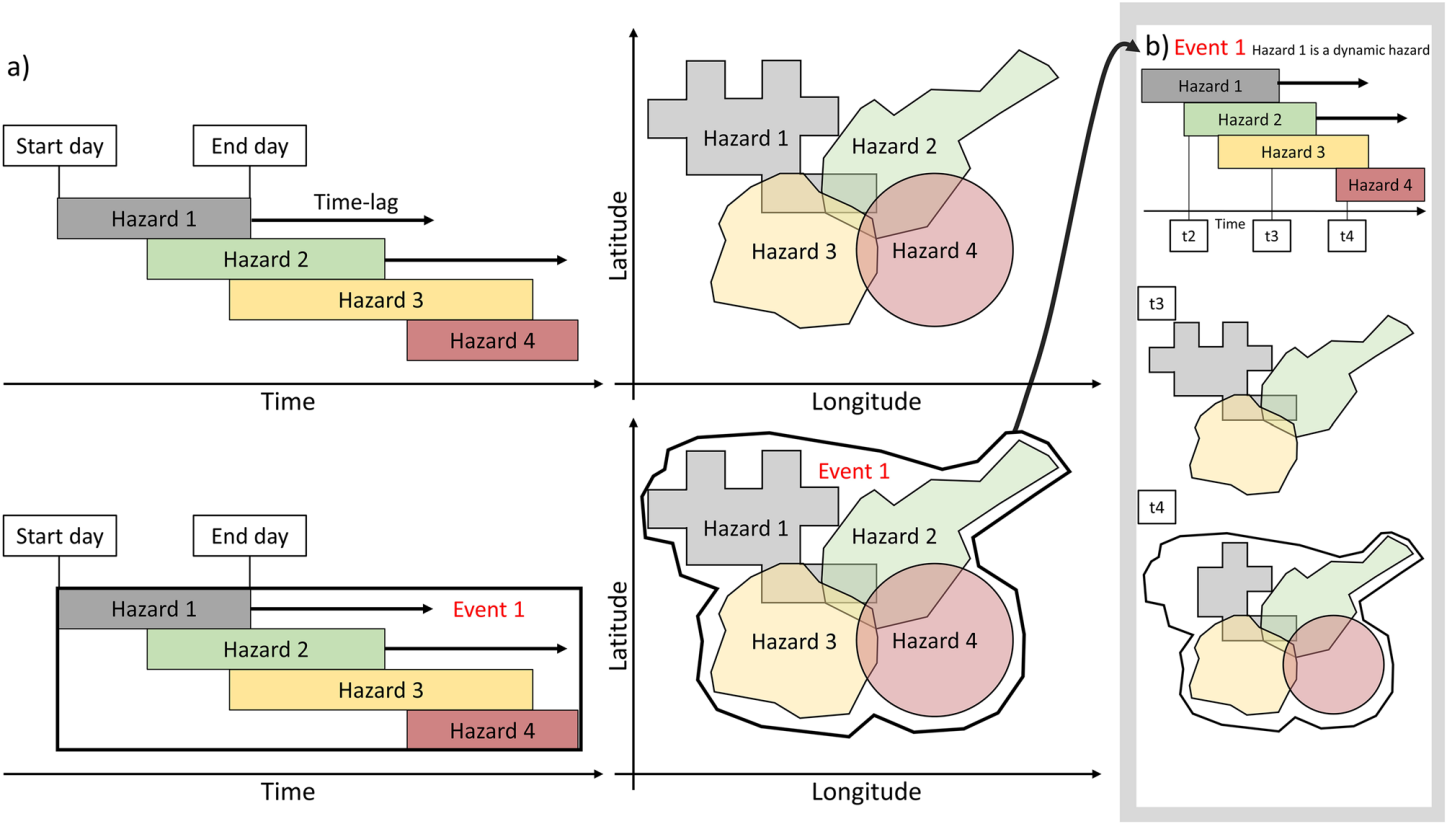
Example of how MYRIAD-HESA operates with a time-lag. (**a**) Two or more hazards are considered a multi-hazard event if they occur at the same location, and at the same time or within each other’s time-lag. Here, there is one hazard group, Event 1, that is encompassed by the black solid line. Event 1 consists of 6 hazard pairs between Hazard 1, 2, 3, and 4 (**b**) The time-lag is also applied to the separate timesteps of a dynamic hazard. Here, Hazard 1 is a dynamic hazard. Therefore, its polygon at timestep 3 (t3) is a combination of its polygons at timestep 1, 2 and 3, when a time-lag of two timesteps has been applied.

To also consider multi-hazard events where the second event occurs after the first one has ended, a time-lag can be introduced (see Fig. 2a). Such a time-lag is the number of days after the first event during which a second event can occur. Two events must overlap spatially to be considered a hazard pair, but no longer have to overlap directly in time. The algorithm enables a time-lag to be introduced for each hazard where multiple hazards are considered a multi-hazard event if they occur within one another’s time-lag. Various time-lags based on the hazard intensity are tested to understand the impact of a time-lag on the number and type of multi-hazard events globally. The time-lag has also been applied to the dynamic hazards, as can be seen in Fig. 1b.

### Single natural hazard datasets

In this paper, MYRIAD-HESA has been exemplified using available hazard data on a global scale. Hazard event data have been collected for eleven different natural hazard types (see Table 1) that have each also been listed in the UNDRR Hazard Definition and Classification review^33^. The datasets used were selected based on their global coverage and available timespan. For several natural hazards, a global footprint database is available, for example, shakemaps for earthquakes. However, for hazards where no global footprint database exists, such as heatwaves, reanalysis products have been obtained. An overview of the data sources used is presented in Table 1.

**Table 1:** Overview of the different data types and sources used in this research, including the intensity units, years available and whether dynamic hazard data is available.

Hazard class	Hazard type	Acronym	Name and source	Intensity unit	Years available	Dynamic hazard
Geophysical	Earthquake	eq	ShakeMaps Earthquake Catalogue^34^	Richter Magnitude [–]	1900–present	No
	Volcanic eruption	vo	Global Volcanism Program^35^	Volcanic Explosivity Index (VEI) [–]	1345 BCE–present	No
	Landslide	ls	Global Fatal Landslide Database^36^	[–]	2004–2017	No
Meteorological	Tropical cyclone	tc	IBTrACS^37^	Saffir-Simpson Scale	1850–present	No
			The Willis Research Network Global Tropical Cyclone Wind Footprint dataset^38^	[ms^−1^]	1989–2020	
	Coldwave	cw	ERA-5^39^	Kelvin	1979–Present	Yes
	Heatwave	hw	ERA-5^39^	Kelvin	1979–Present	Yes
	Extreme wind	ew	ERA-5^39^	[ms^−1^]	1979-Present	Yes
Hydrological	Tsunami	ts	Global Historical Tsunami Database^40^	Wave height [m]	2000 B.C.–present	No
	Flood	fl	The Global Flood Database^41^	[-]	2000–2018	No
Climatological	Drought	dr	Global Drought Observatory^42^	SPI-3	1981–2022	Yes
	Wildfire	wf	Global Wildfire Dataset^43^	[–]	2000–present	Yes

**Figure 3:**
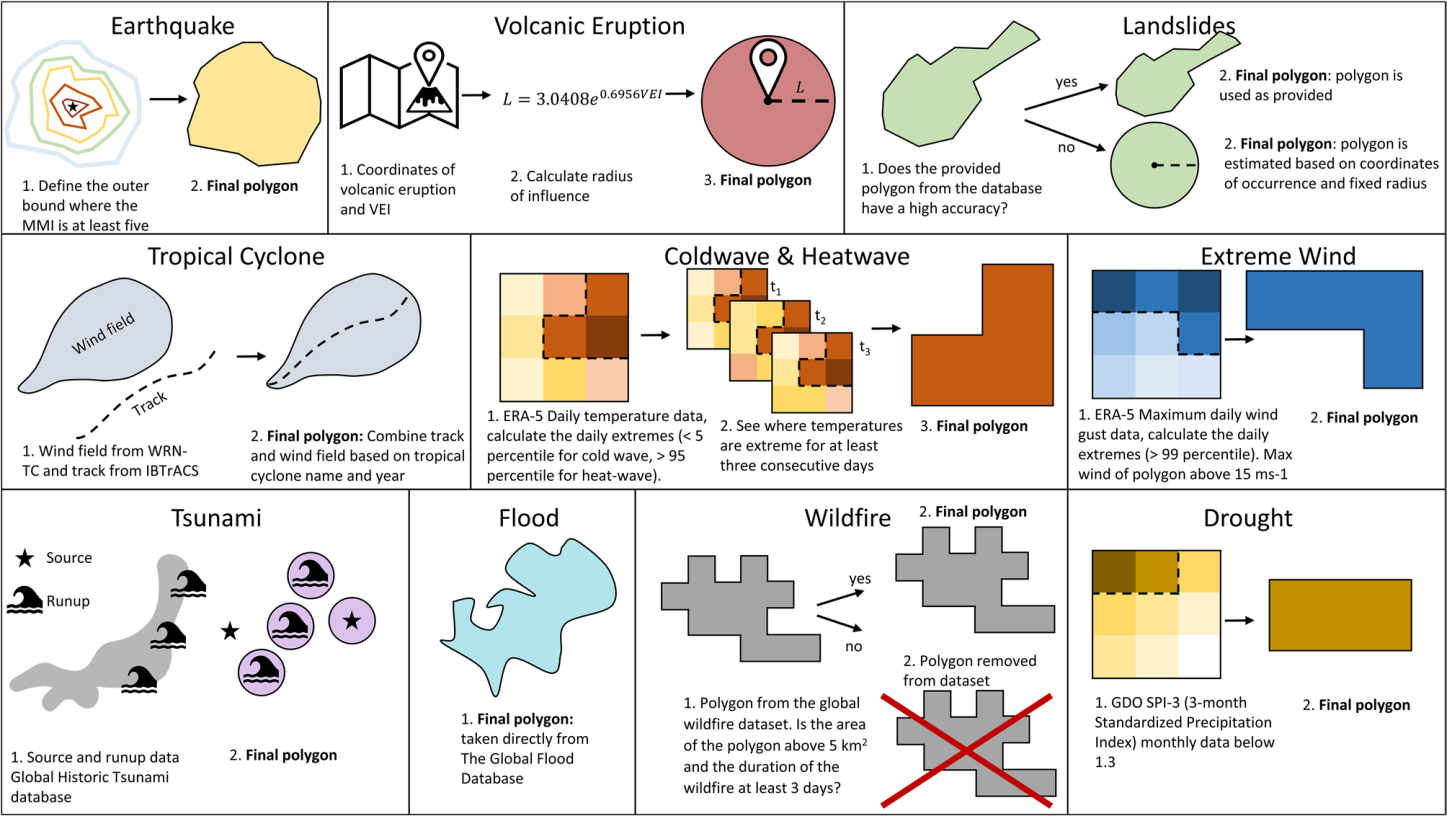
Overview of how the polygons have been defined for each individual hazard.

Below, we discuss the data processing steps taken for each of the hazard types listed in Table 1. A visual summary of how polygons of each natural hazard type have been defined, is presented in Fig. 3. The following subsections can be read in conjunction with both Table 1 and Fig. 3.

#### Earthquake

A record of historic earthquakes has been obtained from the US Geological Survey’s (USGS) Earthquake Catalogue as shakemaps^34^. Shakemaps are automatically generated shaking and intensity maps that combine instrumental data with information about local geology at the location of the event. For this study, only earthquakes with an MMI (Modified Mercalli Intensity) of five or higher have been selected. According to the MMI scale, this is the lowest intensity where damages are expected to occur^44^. For each shakemap, the extent of the event (the event polygon) is based on the outer bound of the area that is hit by an MMI of 5 or higher.

#### Volcanic eruption

A record of volcanic eruptions can be found at the Smithsonian Institution Global Volcanism Program (GVP)^35^. The GVP is a database of all Holocene confirmed eruptions, including their longitude, latitude, start time, end time, and Volcanic Explosivity Index (VEI). The VEI uses the volume of erupted pyroclastic material (ashfall and other ejecta), and the eruption cloud height to assign an intensity value on a scale of 0 to 8^45^. In the *World Atlas on Natural Disaster Risk*^46^ a relationship has been derived between the radius of influence, $L$, and the VEI (see Eq. 1).

1 $$L = 3.0408e^{0.6956VEI}$$

This relationship has been used to create circular event polygons based on the calculated radius of influence.

#### Landslides

Landslide events have been obtained from the Global Fatal Landslide Database that has been developed at the University of Sheffield^36^. As the name suggests, the database only includes landslides that have caused at least one fatality. Key information provided on each landslide includes: the main cause (both human-induced and natural), the longitude, latitude, and a polygon of impact. Some of the polygons have a low precision covering the bounds of an entire country. These polygons have instead been defined as a circular event polygon with the provided longitude and latitude as a center and a radius of 0.2 decimal degrees. The 0.2 degrees are based on the average area of the polygons with higher precision. Furthermore, for the purpose of this study, only the natural hazard-induced landslides have been selected, as so-called NaTech (Natural-Technological)^10^ hazards are outside of the scope of this paper.

#### Tropical cyclones

The tropical cyclone events are obtained from two data sources. Firstly, the International Best Track Archive for Climate Stewardship (IBTrACS), a repository of tropical cyclone tracks. This database includes, amongst other variables, the name, the location of the eye at three or 6-hourly intervals, the maximum wind speeds at those times, as well as the intensity level on the Saffir-Simpson Scale^37^. Secondly, we make use of the Willis Research Network Global Tropical Cyclone Wind Footprint dataset (WRN-TC), with modeled wind footprints based on track information provided by IBTrACS. The event polygons in our analysis have been defined based on the outer bounds of the WRN-TC footprint. Aside from the footprint, WRN-TC only provides the tropical cyclone name and year of occurrence. Based on the name and year, the footprint is linked back to IBTrACS to identify the start time and end time of each tropical cyclone.

#### Coldwave and heatwave

Both the coldwaves and heatwaves have been derived from the ERA5 reanalysis hourly temperature data at 2 m height. This data have been used to derive the daily mean temperature for each grid cell.

A heatwave is generally defined as an above normal temperature for multiple consecutive days^47^. In line with previous studies, we have defined heatwaves as a period where the daily mean temperature at 2 m (above sea-level) is above 95^th^ percentile for three subsequent days or more^20,23,47^. Using the 95th percentile for a particular location and day of the year enables the identification of relatively warmer periods in colder climates and winter months. The event polygon is based on overlapping and adjacent grid cells where the heatwaves occur at the same time. As an intensity indicator, the maximum temperature measured in any of the grid cells included in a polygon is recorded with the event.

Coldwaves have been estimated using a similar approach, where instead of the 95th percentile, the daily temperature has to be below the 5th percentile for three or more consecutive days. Here, the minimum temperature measured in any of the grid cells included in a polygon is recorded with the event.

For both the heatwave and the coldwave events, a cut-off value has been used to reduce the number of events to the most likely disaster. If the heatwave’s maximum temperature is below 0 degrees Celsius, the event is removed. Likewise, if the cold waves minimum temperature is above 0 degrees, it is excluded.

#### Extreme wind

The ERA5 six hourly instantaneous 10 m wind gust data have been used to identify extreme wind events. First, the maximum wind gust per day was calculated. Following that, the 99th percentile was computed per grid cell. A 99th percentile instead of a 95th percentile was selected to obtain the most extreme events. We define an extreme wind event as a day when the maximum wind gust is above the 99th percentile at a particular location. The event polygon is defined based on overlapping and adjacent grid cells where the 99th percentile is exceeded at the same time. Overlapping event polygons on consecutive days are combined, and the start date is the first day on which the extreme wind is recorded, while the end date is the last day on which extreme wind is recorded. For the extreme wind event, the maximum wind speed has to be above 15 ms^−1^ to be included. This cut-off value is selected to limit the number of events to those with a larger likelihood of being hazardous. While wind is also an element of the tropical cyclone data, it has been included in this study to represent additional extreme wind events that are not classified as tropical cyclones, such as winter storms.

#### Tsunami

The tsunami events in the Global Historic Tsunami database include the date, cause, magnitude of associated earthquakes, longitude and latitude, maximum water height, and the number of runups^40^. The number of runups refers to the location where tsunami effects occurred on shore, which is available in an additional dataset. The runup locations have been linked to the tsunami source, the point where the tsunami originated from. Both the source and the runup locations are relevant to include, as the source can overlap with the hazard that caused the tsunami, such as an earthquake, and the runup locations may overlap with further hazards on land, such as triggered landslides. Coordinates of the runup locations and the tsunami sources are used to create an estimate of a single event polygon by adding a buffer of 1 decimal degree (approximately 111 km) to each location.

#### Flood

The Global Flood Database includes an estimate of flood extent for large flood events from 2000 to 2018 with the use of satellite imagery^41^. The inundated area of each event has been used to create the event polygon. There are a total of 913 flood events in this dataset.

#### Drought

Drought events have been obtained from the Global Drought Observatory’s (GDO) SPI-3 (three-month Standardized Precipitation Index) global database^42^. The GDO provides the SPI-3 at a monthly resolution. According to the definition used by Ridder et al.^23^, a drought is defined when the SPI-3 is below − 1.3. The final event polygon is defined based on overlapping and adjacent grid cells where the SPI-3 is low in consecutive months.

#### Wildfire

The wildfire events in the global wildfire dataset have been obtained through a data-mining approach using NASA’s MODIS burnt area product Collection 6 (MCD64A1)^43,48^. The global burned area products, derived from satellite imagery, provide information on spatial and temporal attributes of all areas affected by fires, but they do not contain information on single wildfire events. Therefore, Artés et al.^43^ used a clustering algorithm to combine single day wildfires into multi-day wildfire events if the fire was active on consecutive days. As the dataset includes millions of events, only those that are above 5 km^2^ in final polygon size are considered in our analysis. This increases the likelihood that an event is actually a wildfire, as MODIS can also registers industry as a fire.

## Results and discussion

### Multi-hazard pairs

**Figure 4:**
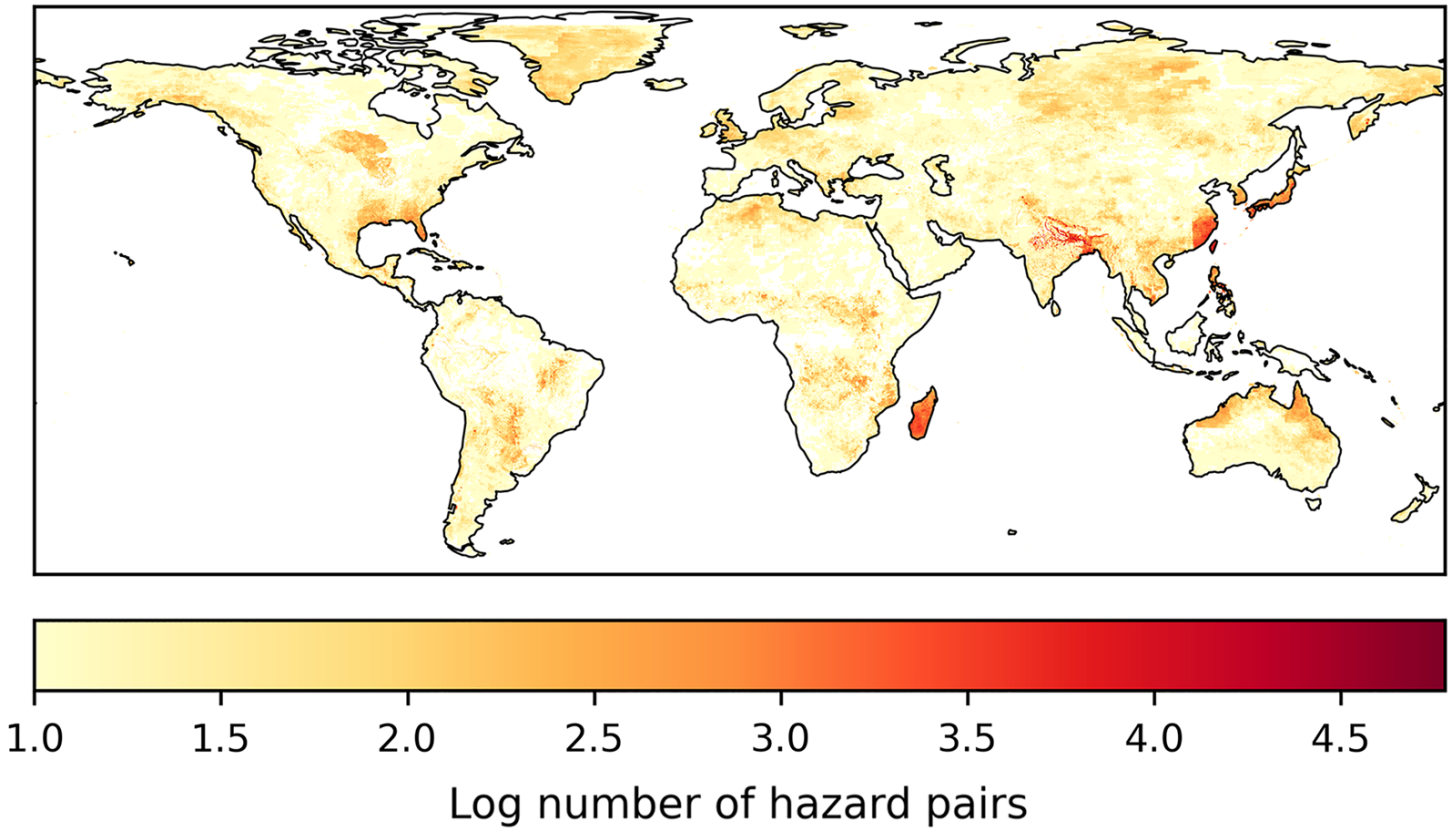
The total number of hazard pairs on a logarithmic scale between 2004 and 2017 showing the hazard pair hotspots globally. White areas are the ocean or a place with no hazard pairs.

Figure 4 shows the global hotspots of hazard pairs in the data without a time-lag. Some notable areas, with a large amount of hazard pairs, include northeast India, Bangladesh, East China, Taiwan, Japan, parts of Southeast Asia, Madagascar, southeast USA, UK, and northern Australia. There are noticeably also locations where no hazard pairs were registered based on the data used, such as in central Africa and the north-central part of South America. This does not mean there are no natural hazards at these locations, rather that there was no hazard overlap detected in our compiled event sets based on historical records.

**Figure 5:**
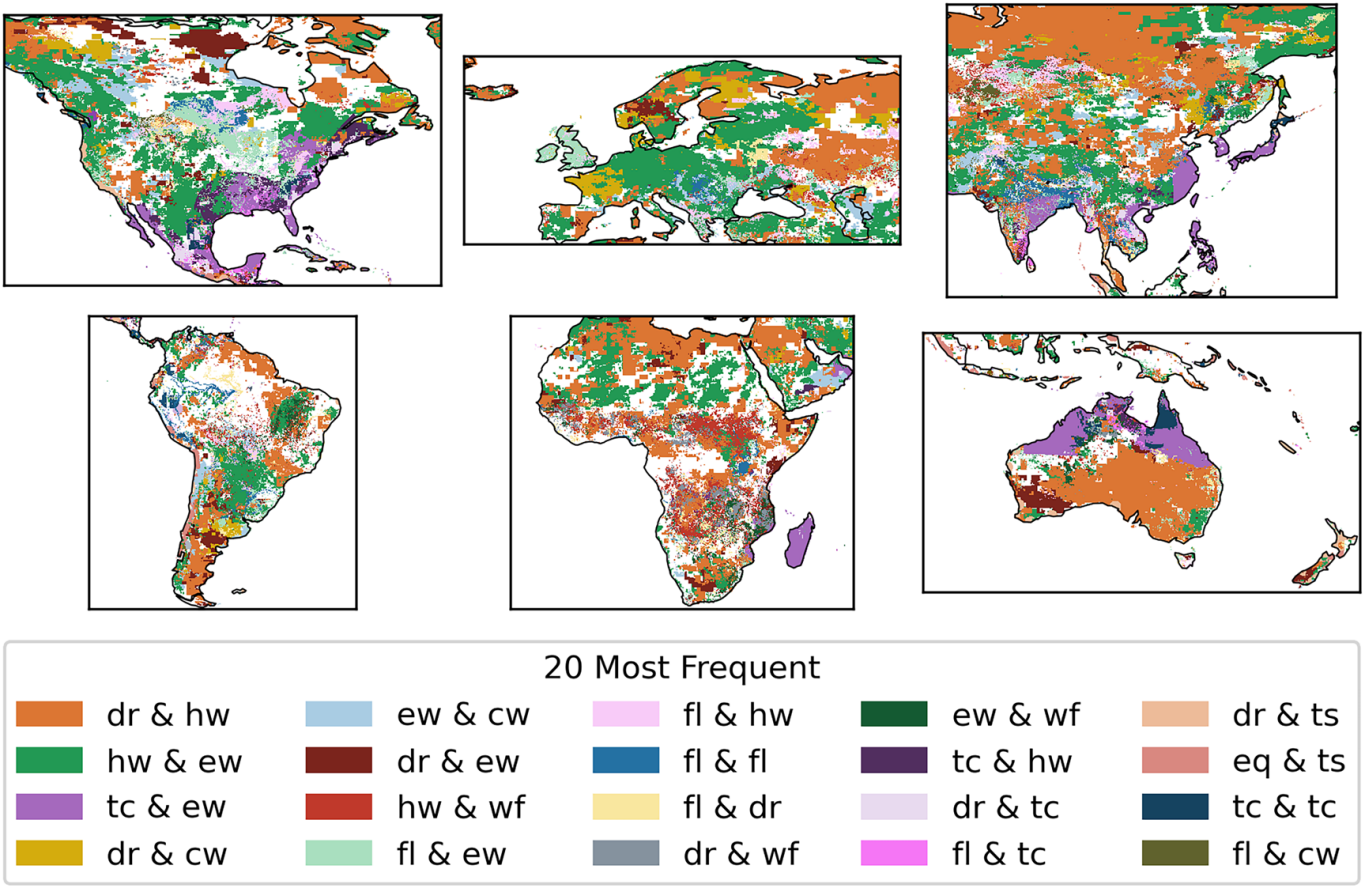
The most frequent hazard pair globally. Here, there is no distinction which hazard occurs first in the pair, for example, ‘dr & hw’ could be a drought followed by a heatwave as well as a heatwave followed by a drought. The acronyms for each hazard are inlcuded in Table 1. White areas are the ocean or a place with no hazard pairs.

The two most prominent hazard pairs globally are the combination of droughts and heatwaves as well as the combination of heatwaves and extreme wind (See Fig. 5 and Supplementary Table 1). The link between drought and heatwaves is evident, as high temperatures can lead to dry conditions and dry conditions can further increase temperatures. Moreover, the combination of a drought and a heatwave is a typical compound event that has received much attention in the past years as they usually lead to severe impacts on socioeconomic factors, are widespread, and are likely to intensify under climate change^20,26,49–51^. In contrast, the link between heatwaves and extreme wind is less evident in literature. The frequent overlap between heatwaves and extreme wind could be due to the relationship between rising hot air and convective storms that occur in summer. However, the frequent overlap may also be caused by the nature of the data. As explained in section “Coldwave and heatwave”, heatwaves are based on above average temperatures of specific calendar days. This means that heatwaves during, for example the European winter storm season, may not necessarily be a typical ‘hot’ summer day. An extreme example of such a winter heatwave are the unprecedented temperatures Europe experienced in January 2023, where the temperature was 10 C^o^ above the average and records were broken by 4 °C^52^. A seasonality analysis of the overlap between heatwaves and extreme wind reveals that the majority of pairs occur during the winter season (see Supplementary Fig. 1). Therefore, this overlap likely represents above average temperature winter days in combination with wind storms, and not summer heatwaves that drive convective storms.

The hazard pair that occurred the most between 2004 and 2017, varies strongly between the various geographic locations (see Fig. 5). Hotspots (as shown in Fig. 4) are often dominated by a combination of tropical cyclones and extreme wind. This is to be expected as tropical cyclones are defined by their high wind speed. While extreme wind and tropical cyclones are not technically two separate hazards, extreme wind was included to also represents storms that are not tropical cyclones. A clear overlap between the two indicate that extreme wind could be a reasonable proxy for storm data.

Additionally, a more spatially scattered, but prominent hazard pair is that of wildfires and heatwaves. This pair can predominately be observed in sub-Saharan Africa, known as the African savannah fires. While indeed more than half of the burned area globally occurs in the African savannahs, it should be noted that these are often human-ignited, a fire source that is difficult to distinguish in the data^53^. The pair is also prominent in South America, near the Amazon as well as in Portugal, Australia, Eastern Europe and Russia.

We observe many pairs that include a flood, such as in the UK where the combination between floods and extreme wind are the most frequent hazard pair. Here, the extreme wind event is likely a storm that is paired with storm surge and/or extreme precipitation, commonly referred to as a compound flood. Various research has shown that these compound floods occur most frequently as a consequence of European winter storms^54,55^, however, they can also occur during summer with devastating impacts, as has been observed during the July 2021 UK floods^56^. Likewise, floods are prominent in Bangladesh, in a flood to flood hazard pair. This is not surprising as 80% of Bangladesh is a flood plain and the largest number of people affected due to a natural hazard in Bangladesh since 1972 can be attributed to floods^57^.

Furthermore, tsunami-related pairs are visible on the coastlines for California (U.S.A), New Zealand, Japan, and Sumatra (Indonesia). They are often paired with earthquakes, as can be expected since earthquakes are the main cause of tsunamis, but they are also coinciding with droughts, which is more surprising. We suspect there is likely no link between the two, and the frequent overlap occurs due to the large spatial scale and the duration of a drought (see supplementary Table 2).

**Figure 6:**
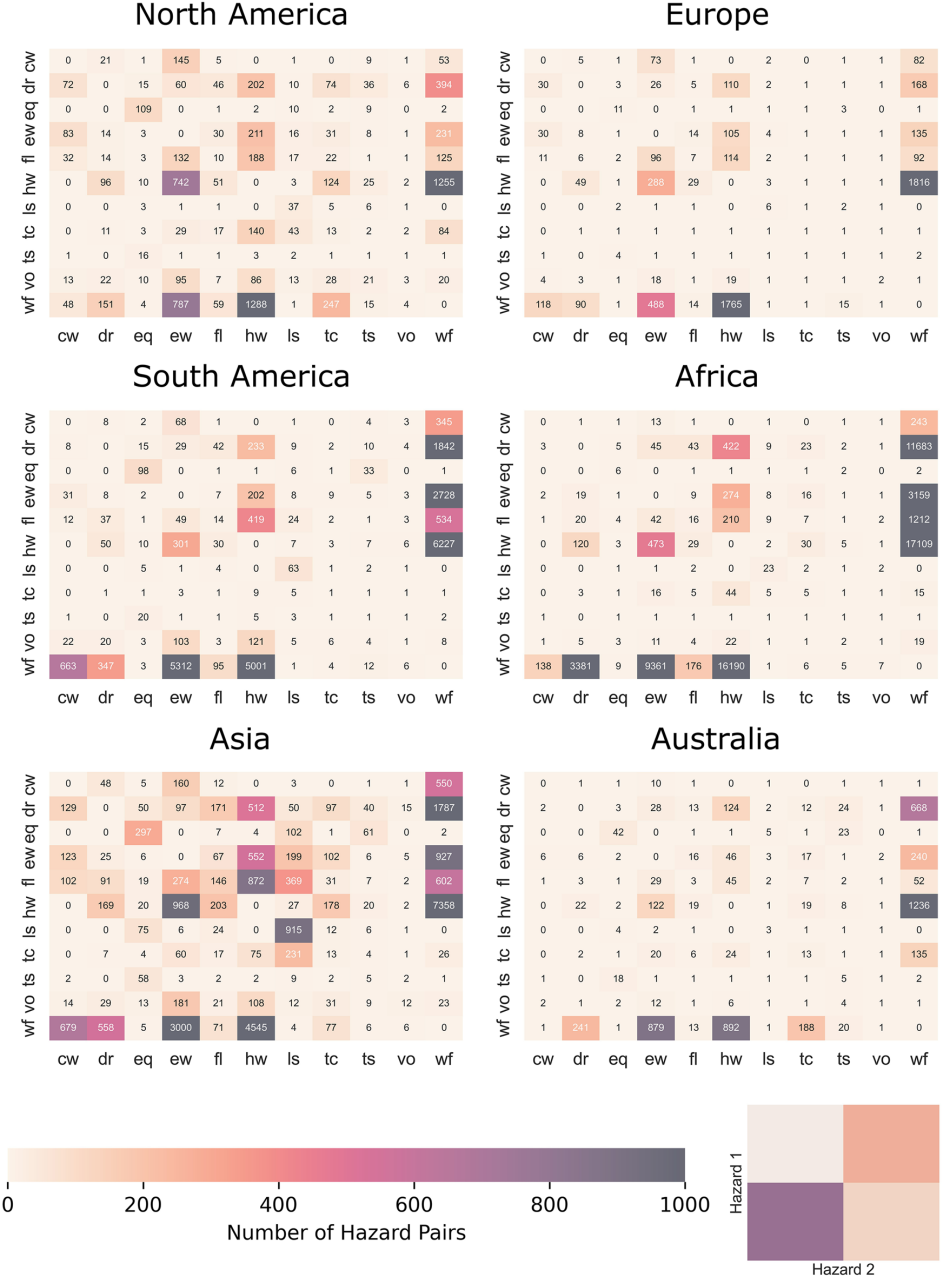
Heatmaps showing the number of hazard pairs per continent. Hazard 1 is the hazard that has started first, followed by Hazard 2. The acronyms for each hazard are inlcuded in Table 1. The total number of hazard pairs identified is 13,764. The different continents are represented in the Supplementary materials Fig. 2.

As Fig. 5 only shows the most frequent hazard pair spatially, it does not show all possible pairs and their frequency. Therefore, the total number of unique hazard pairs per continent is provided in Fig. 6. Here, a couple of interesting observations stand out. Firstly, there are a high number and a large variety of hazard pairs in Asia, most notably in comparison to Europe and Australia. This difference may be due to the size of each continent, but also its geographic and diverse topography. Most notably landslides appear significantly more in Asia compared to other continents. This may be due to a registration bias, as reporting on landslides tend to be for those with the largest impacts. For example, EM-DAT shows that approximately 54% of the registered high-impact landslides between 1910 and 2022 occurred in Asia. On the other hand, landslides that occur in remote regions with relatively smaller amounts of impacts are generally not reported, and it could be that there are more urbanised areas susceptible to landslides in Asia^36,58,59^.

Secondly, there are many pairs that include wildfires. This is because wildfires are the most frequent individual hazard event type in the database (see Supplementary Table 2). Similarly, the single hazards derived from the ERA-5 data are abundant in pairs due to their high amount of global data availability.

Overall, the heatmaps show that there is a large variety of hazard pairs possible globally and that the secondary hazard (Hazard 2), can be preceded by a variety of initial hazards (Hazard 1). This is illustrated well by the columns where landslides are the secondary hazards, for example in Asia. The landslides are often a second hazard following an earthquake, flood, extreme wind, or a tropical cyclone. There is also a large overlap between landslides, possibly due to the same trigger, or a primary landslide initiating a secondary landslide. The connection between landslides and their possible trigger can be better understood by the hazard groups described in the next section.

### Multi-hazard groups

In addition to hazard pairs, hazards can overlap in larger numbers as hazard groups (see Fig. 2). Between 2004 and 2017, 131,318 hazard groups have been identified. Hazard groups are listed based on order of occurrence of the individual hazard, meaning that the hazard with the earliest start date is first in the list. Of these groups, 485 original hazard combinations were determined. The original hazard groups vary greatly in frequency of occurrence, from 1 to 33,381 times, where an occurrence of 1 means that the particular order of hazards has only occurred once.

**Figure 7:**
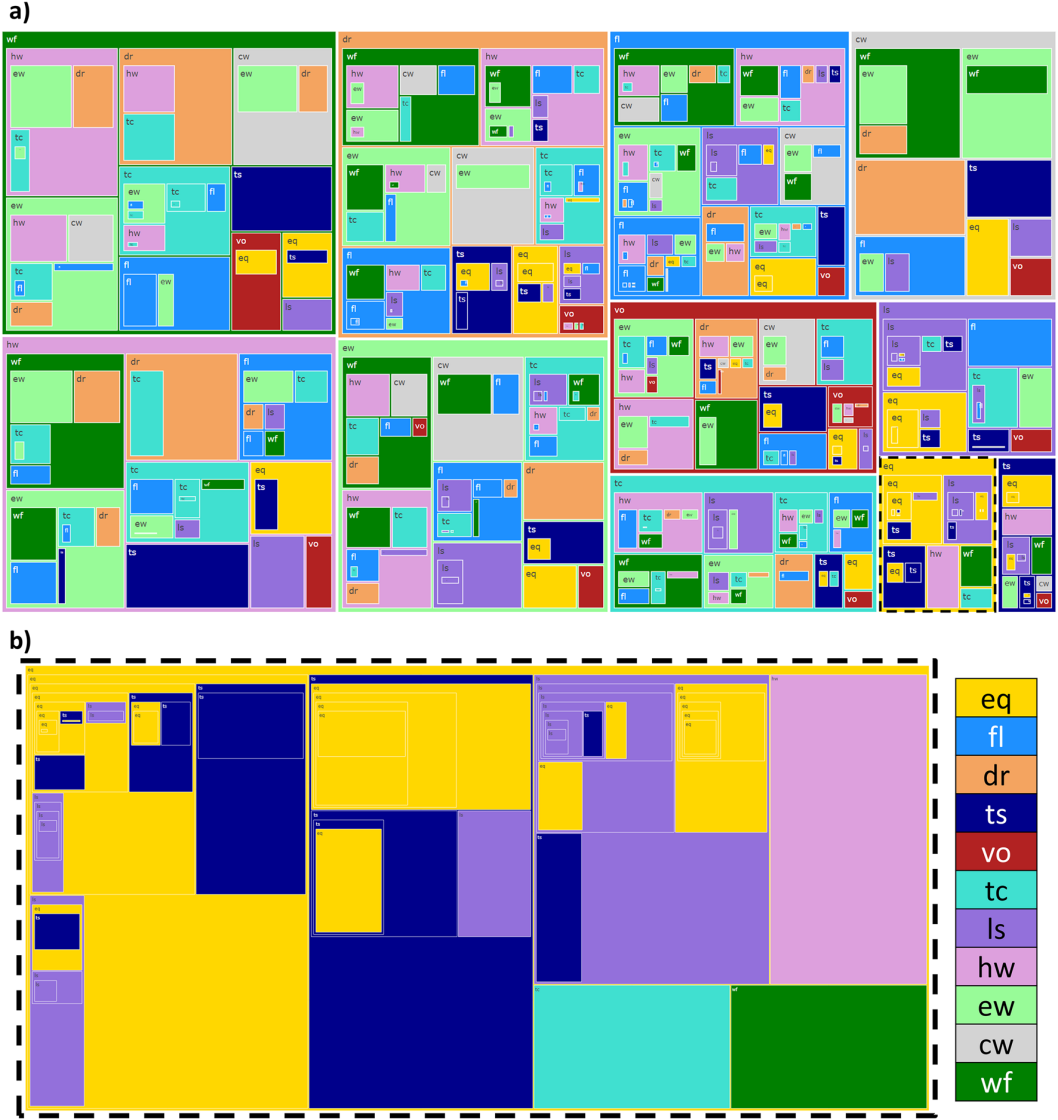
(**a**) Tree-map of all the unique hazard groups and their frequency of occurrence. The outer rectangles represent the first hazard in a group. The nested rectangles within represent the following hazards in the group, where each level in the hierarchy is the next hazard in the list, from hazard_1_ to hazard_n_. The rectangles are sized based on the frequency of occurrence. However, because some groups occurred four orders of magnitude more, the box sized have been weighted to be able to properly visualize the less frequent groups as well. This implies that the size is not directly proportional to the frequency, but the sizes of the rectangles at each hierarchy level are ordered from large to small for higher to lower frequencies respectively. Therefore, the most frequent hazard is still represented by the largest box. (**b**) A zoom into the hazard groups that have an earthquake as first hazard. The acronyms for each hazard are inlcuded in Table 1. The interactive treemap is available as a .html file in the supplementary material. The interactive tree map allows to zoom into different hazard groups and includes quantitative information on the frequency of occurrence of each group.

Figure 7 shows all unique hazard combinations and the frequency of occurrence. The majority of the groups have a wildfire as a first hazard, while the lowest number of groups has a tsunami as a first hazard. Across all groups, most hazard groups do consist of only two hazards, a hazard pair. However, groups of three hazards are also not uncommon. The largest groups predominately occur with an earthquake as a first hazard (Fig. 7b). The largest group has eleven hazards in it, and consists of three earthquakes and nine landslides. This is partially due to overlapping earthquakes that could be an initial earthquake and its aftershocks. Large earthquakes with many aftershocks are also a known cause for tsunamis, as seen in the tree map. Other large groups include many landslides. These landslides could all be triggered by the same earthquake or have triggered one another as a consequence of slope instability caused by an initial landslide, as discussed in the previous subsection. These results reflect those of Gill and Malamud^60^, where different hazard interactions were identified. Here it is noted that an earthquake can trigger a multitude of landslides and that a landslide can trigger and increase the probability of a secondary landslide.

### Time-lag

In the previous section we assumed that hazards have to overlap in both space and time to form a pair or group. However, the impacts of two, or more, hazards can also be interrelated through time in-between hazards. Therefore, it is of interest to investigate how the multi-hazard events respond to a time-lag between hazards. In this section, North America serves as a case study to illustrate the impact of a time-lag.

**Figure 8:**
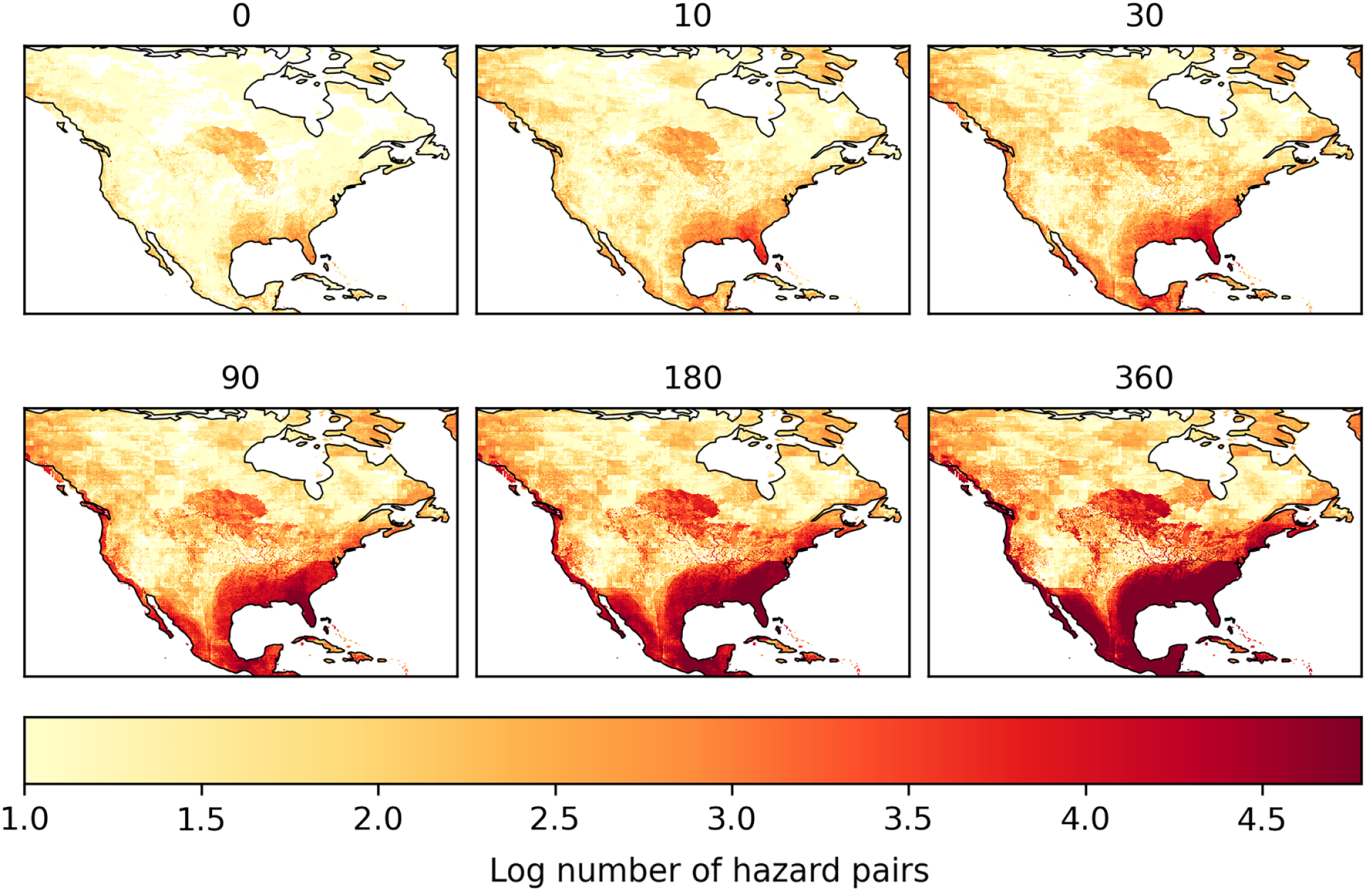
The number of hazard pairs in the US with varying time-lags (in days) on a logarithmic scale.

By definition, a larger time-lag between hazards results in more hazard pairs, as each hazard will have a larger time frame in which hazards can overlap. This is also evident in the total number of hazard pairs in the United States (Fig. 8). The relative increase between different time-lags appears to be larger in the first 10 to 30 days, compared to the difference between 180 and 360 days (see Supplementary Fig. 3). Furthermore, the major hotspots remain similar through time. While the hotspot does expand in the south of the continent, Florida remains the region with the largest number of hazard pairs.

**Figure 9:**
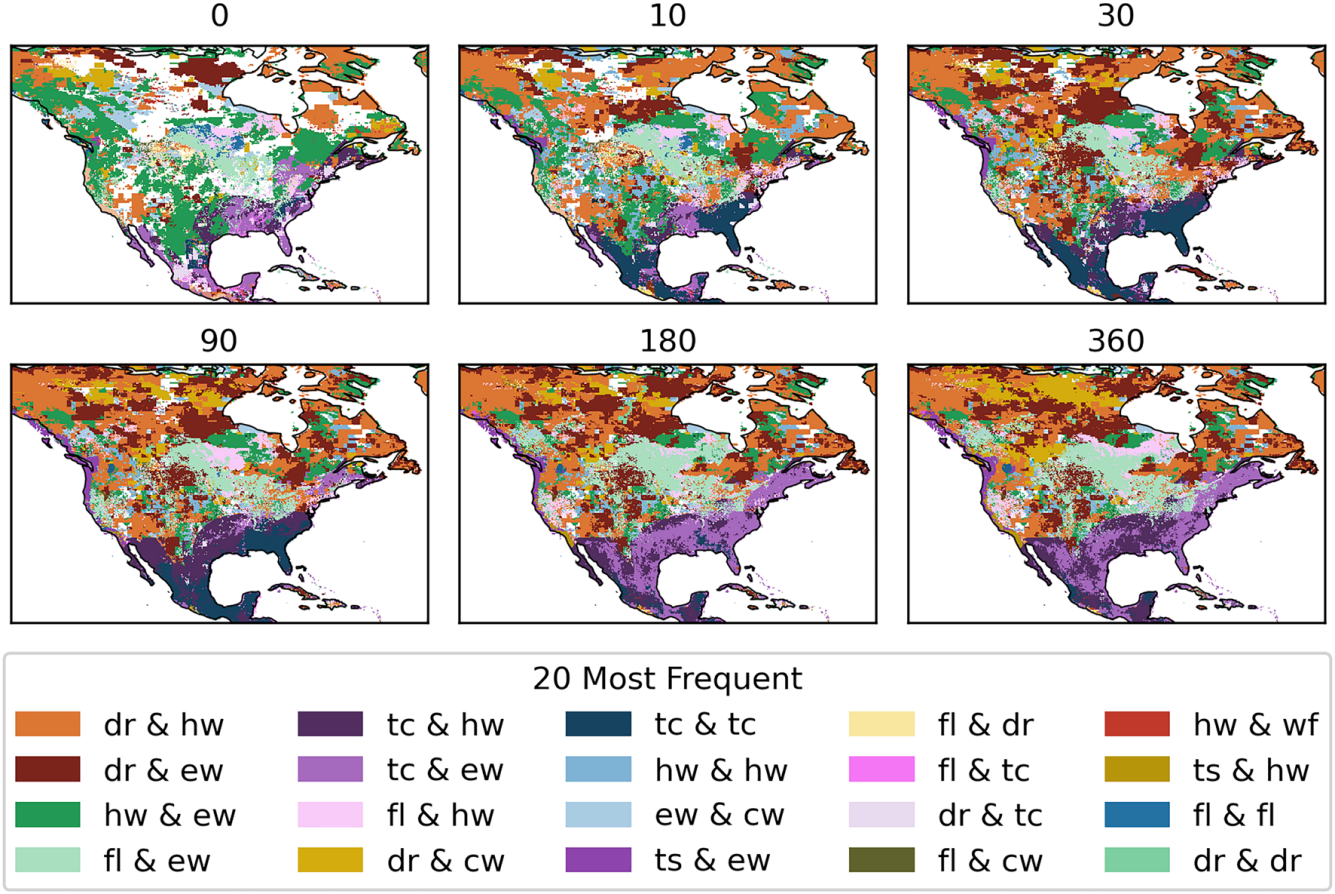
The most frequent hazard pairs in North America with varying time-lags from 0 to 360 days. The acronyms for each hazard are inlcuded in Table 1.

To better understand the hotspots with varying time-lags, the most frequent hazard pair at each location are shown in Fig. 9. Here it is clear that the south of the continent is dominated by tropical cyclone related hazard pairs, regardless of the time-lag. However, the hazard that is paired with the tropical cyclone does vary. A time-lag of 0 to 10 days still shows spots of overlap with floods, a known consequence of tropical cyclones. This hazard pair is relatively less frequent, in comparison to other hazard pairs, with larger time-lags as the flood usually occurs during the tropical cyclone event, or shortly after. Time-lags between 10 and 90 days show that tropical cyclones overlap with themselves most frequently. This can be explained by the Atlantic hurricane season, which runs from June to November. A notable hurricane season included in this database occurred in 2004. For the first time in the US hurricane record, four hurricanes hit Florida in close succession, namely, Hurricane Charley in August followed by Frances, Ivan and Jeanne in September. Frances and Jeanne hit the same coast at virtually the same location, which had also not been recorded before during the same season. While the secondary hazard, hurricane Jeanne, was not as intense compared to hurricane Frances, Jeanne caused leftover storm debris to fly around, and further tear apart already weakened buildings. Hence Jeanne likely caused more damage than it would have if it occurred in isolation. Furthermore, it was difficult to attribute total damages to the individual hurricanes^61,62^. Attributing damages is a common challenge when hazards occur in close succession as consecutive hazards. For example, attributing all further damages to the secondary hazard may lead to an incorrect damage assessment, while incorporating a time-lag to observe hazards that occurred previously can help understand how a hazards of a particular magnitude managed to cause the observed damages^10^.

Following the hurricane season, no more overlaps between tropical cyclones were registered with a time-lag beyond the duration of the season. This allows other hazard pairs to become more frequent with a time-lag of 180 to 360 days. The more frequent hazard pair with longer time-lags are between tropical cyclones and extreme wind or heatwaves, as these events occur all throughout the year, hence will be registered with an additional time-lag.

The remainder of North America shows a general expansion of many hazard pairs that already occurred with no time-lag, such as a heatwave and a drought, a flood and extreme wind, a drought and extreme wind, a heatwave and extreme wind, as well as a flood and a heatwave.

### Implications of the methodology

While MYRIAD-HESA aims to successfully incorporate hazards from varying hazard classes into one database, our results show that the difference in how each of the hazard footprints has been defined strongly influences the resulting hazard pairs and groups. The most frequent groups/pairs often include the hazards derived from ERA-5 data, wildfires (based on the MODIS product) and/or droughts (based on SPI-3). These all have good global coverage and are based on thresholds, resulting in a large number of small events. Comparatively, observation-based data, such as landslides and tsunamis, have fewer individual hazard events. Hence, they occur in fewer multi-hazard pairs/groups. Additionally, the size and duration of each event also impacts the number of hazard pairs. Each natural hazard occurs on varying spatial and temporal scales (see Fig. 1 of Gill and Malamud^60^). Tropical cyclones are spatially large weather systems, while not being nearly as frequent as, for example, a wildfire. Therefore, a tropical cyclone will have more hazard overlaps compared to hazards with a typically smaller spatial footprint, such as a volcanic eruption. Likewise, hazards with a longer duration, such as droughts that have an average duration of 61 days, will have a higher likelihood to overlap with a secondary hazard (see Supplementary Table 2).

As we expected, the specified time-lag between hazards influences the resulting multi-hazard events. Both in the frequency of different hazard pairs as well as the number of multi-hazard pairs/groups. This time-lag, while only hypothetical, is crucial to identify consecutive hazard events where there were potential interrelated impacts, such as the four hurricanes that hit Florida in 2004. In our study, the same time-lag was used for all hazards of all intensities, however, the method can also be adjusted to have varying time-lags for different hazards and intensities. For example, an earthquake with MMI 10 may need a longer time-lag compared to an earthquake of MMI 5, as it likely caused more damage and can influence the impact of a secondary hazard.

Finally, while the results presented in this paper provide an insight in potential multi-hazard events on a global scale, these findings are limited by the data coverage. The lack of country-level data creates an inability to capture small events, such as flash floods that are generally not included in the global flood database, or landslides that were not fatal but did cause damage. Likewise, the global gridded data used in this study, such as the ERA5 reanalysis data, do not provide a sufficient resolution to capture small events in comparison to local data. Additionally, the assumptions used to define the hazard events from reanalysis data may lead to an over or underestimation of the number of events, as there is no guarantee that these were hazardous. Furthermore, the representation for volcanic eruptions is based on the potential area of impact due to pyroclastic and lava flows. Therefore, the negative impacts of ash and gasses, which operate on a larger spatial scale, are ignored. Hence, fewer multi-hazard events including volcanic eruptions are identified compared to reality. However, the events in MYRIAD-HES can serve as a guide to identify events that had severe consequences, which is a key first step in understanding the complex multi-hazard interactions that drive impacts. Additionally, MYRIAD-HESA can further overcome data limitation by allowing the user to incorporate their own higher resolution data for an area of interest, and the results can easily be altered if data quality improves with future innovations (see code availability). However, it should be noted that in order to incorporate new data, the data needs to be formatted as polygons, such as those represented in Fig. 3.

## Conclusions and applications

With the use of historic global hazard data, this study provides a primary multi-hazard event set, MYRIAD-HES, that has been created with a new method, MYRIAD-HESA. We show that single hazard data from varying sources can be formatted and combined into a multi-hazard dataset, even if the hazards occur on varying time, space, and intensity scales.

MYRIAD-HES is presented to identify global hotspots of hazard pairs, which hazard pair occurs most frequently in different regions, and unique multi-hazard groups with and without time-lag. The most frequent hazard pair globally is the combination between heatwaves and droughts. However, most of the global hotspots, such as Madagascar, Florida, North Australia, Bangladesh, Japan and the Philippines, are largely dominated by tropical cyclone activity and resulting secondary hazards, such as floods and landslides. The hazard groups resulting from the hazard pairs can be very complex, as large numbers of variations are possible. The grouped events can consist of many different hazards, making it more difficult to analyse compared to hazard pairs. However, it is evident in the multi-hazard groups that the larger groups often include a cascade of many landslides. Finally, a time-lag has been introduced to observe hazards that occur at the same spatial locations with a set amount of time in between. Here, North America served as a case study to show how varying time-lags from 0 to 360 days bring to light varying multi-hazard interactions. This is of importance to highlight scenarios where two impactful events hit in close succession, such as the hurricanes that hit Florida in 2004, and the tropical cyclone that followed the earthquake in Haiti 2021.

MYRIAD-HESA may be of interest to a variety of practitioners. This method allows governments to plan for specific multi-hazard events and provide insights into the number of overlapping events they need to account for. NGOs can consider the information on locations of global hotspots that are susceptible to multi-hazards to prioritise the allocation of (future) resources. (Re-)insurers could benefit from using this multi-hazard approach by recognizing their assets may be at risk of multiple hazards at the same time, hence making more informed decisions. Finally, this method is open-source and can therefore be used in further research to advance understanding of multi-hazard events by incorporating improved hazard data, hazard relationship insights, and damage information. For example, the multi-hazard footprints could be linked to disaster databases, such EM-DAT^59^, to compliment the impact data with additional hazard information as well as to identify which other hazards have hit the sites in close succession. This information will enable the user to evaluate what impact additional hazards may have had on the magnitude of the damages.

### Supplementary Information

Supplementary Information 1.

Supplementary Information 2.

## Data Availability

MYRIAD-HES, the dataset compiled during the is study, is openly available on Zenodo^63^.

## Code availability

The MYRIAD-HESA code has been publicly released on GitHub^32^.

## References

[1] Martinez, S. N. *et al.**Landslides Triggered by the August 14, 2021, Magnitude 7.2 Nippes, Haiti, Earthquake*. Open-File Report (2021).

[2] Quigley MC, Attanayake J, King A, Prideaux F (2020). A multi-hazards earth science perspective on the COVID-19 pandemic: The potential for concurrent and cascading crises. Environ. Syst. Decis..

[3] UNDRR. Report of the open-ended intergovernmental expert working group on indicators and terminology relating to disaster risk reduction. **21184**, 1–41 (2016).

[4] Zschau, J. Where are we with multihazards, multirisks assessment capacities? *Sci. Disaster Risk Manag.* 98–115 (2017).

[5] UNDRR. *UNDRR: Sendai Framework for Disaster Risk Reduction 2015–2030* (2015).

[6] Pörtner, H. *et al. Climate Change 2022: Impacts, Adaptation and Vulnerability Working Group II Contribution to the IPCC Sixth Assessment Report Citations to the Sixth Assessment Report of the Intergovernmental Panel on Climate Change [to the Sixth Assessment Report]*. 10.1017/9781009325844.Front (2022).

[7] Ward PJ (2022). Invited perspectives: A research agenda towards disaster risk management pathways in multi-(hazard-)risk assessment. Nat. Hazard. Earth Syst. Sci..

[8] Terzi S (2019). Multi-risk assessment in mountain regions: A review of modelling approaches for climate change adaptation. J. Environ. Manag..

[9] de Angeli S (2022). A multi-hazard framework for spatial-temporal impact analysis. Int. J. Disaster Risk Reduct..

[10] de Ruiter MC (2020). Why we can no longer ignore consecutive disasters. Earth’s Future.

[11] Zscheischler J (2020). A typology of compound weather and climate events. Nat. Rev. Earth Environ..

[12] de Ruiter MC, van Loon AF (2022). The challenges of dynamic vulnerability and how to assess it. iScience.

[13] Zscheischler J, Raymond C, Horton RM, Ramos AM (2020). A typology of compound weather. Nat. Rev. Earth Environ..

[14] Gill JC, Malamud BD, Barillas EM, Noriega AG (2020). Construction of regional multi-hazard interaction frameworks, with an application to Guatemala. Nat. Hazard..

[15] Tinti S, Pagnoni G, Piatanesi A (2003). Simulation of tsunamis induced by volcanic activity in the Gulf of Naples (Italy). Nat. Hazards Earth Syst. Sci..

[16] Kew SF, Selten FM, Lenderink G, Hazeleger W (2013). The simultaneous occurrence of surge and discharge extremes for the Rhine delta. Nat. Hazards Earth Syst. Sci..

[17] Van Den Hurk B, Van Meijgaard E, De Valk P, Van Heeringen KJ, Gooijer J (2015). Analysis of a compounding surge and precipitation event in the Netherlands. Environ. Res. Lett..

[18] Wang J, Gao W, Xu S, Yu L (2012). Evaluation of the combined risk of sea level rise, land subsidence, and storm surges on the coastal areas of Shanghai. China. Clim Change.

[19] Xu K, Ma C, Lian J, Bin L (2014). Joint probability analysis of extreme precipitation and storm tide in a coastal city under changing environment. PLoS ONE.

[20] Sutanto SJ, Vitolo C, di Napoli C, D’Andrea M, van Lanen HAJ (2020). Heatwaves, droughts, and fires: Exploring compound and cascading dry hazards at the pan-European scale. Environ. Int..

[21] Moftakhari HR, Salvadori G, AghaKouchak A, Sanders BF, Matthew RA (2017). Compounding effects of sea level rise and fluvial flooding. Proc. Natl. Acad. Sci. U. S. A..

[22] Tilloy A, Malamud BD, Joly-Lauge A (2021). Earth Syst. Dyn..

[23] Ridder NN (2020). Global hotspots for the occurrence of compound events. Nat. Commun..

[24] Li D (2022). Compound wind and precipitation extremes across the Indo-Pacific: Climatology, variability and drivers. Geophys. Res. Lett..

[25] Owen LE, Catto JL, Stephenson DB, Dunstone NJ (2021). Compound precipitation and wind extremes over Europe and their relationship to extratropical cyclones. Weather Clim. Extremes.

[26] Zscheischler J (2018). Future climate risk from compound events. Nat. Clim. Change.

[27] Raymond C (2020). Understanding and managing connected extreme events. Nat. Clim. Change.

[28] Marzocchi W, Garcia-Aristizabal A, Gasparini P, Mastellone ML, Di Ruocco A (2012). Basic principles of multi-risk assessment: A case study in Italy. Nat. Hazards.

[29] Gill JC (2021). Invited perspectives: Building sustainable and resilient communities-recommended actions for natural hazard scientists. Nat. Hazard..

[30] Peduzzi P (2019). The disaster risk, global change, and sustainability nexus. Sustainability (Switzerland).

[31] Tilloy A, Malamud BD, Winter H, Joly-Laugel A (2019). A review of quantification methodologies for multi-hazard interrelationships. Earth-Sci. Rev..

[32] Claassen, J. judithclaassen/MYRIAD-HESA: MYRIAD – Hazard Event Sets Algorithm (MYRIAD-HESA). 10.5281/ZENODO.8272755 (Zenodo, 2023).

[33] UNDRR. Hazard definition & classification review: Technical report. Hazard Definition Classif. Rev. Defin. Classif. Rev. 1–88 (2020).

[34] US Geological Survey (2017). ShakeMap – Earthquake ground motion and shaking intensity maps. U. S. Geol. Survey.

[35] Global Volcanism Program. *Choice Reviews Online,* Vol. 45. 10.5479/si.GVP.VOTW4-2013 (2013).

[36] Froude MJ, Petley DN (2018). Global fatal landslide occurrence from 2004 to 2016. Nat. Hazard..

[37] Knapp KR, Kruk MC, Levinson DH, Diamond HJ, Neumann CJ (2010). The international best track archive for climate stewardship (IBTrACS). Bull. Am. Meteorol. Soc..

[38] Done JM (2020). Modelling global tropical cyclone wind footprints. Nat. Hazards Earth Syst. Sci..

[39] Copernicus Climate Change Service. ERA5: Fifth generation of ECMWF atmospheric reanalyses of the global climate. https://cds.climate.copernicus.eu/cdsapp#!/home (2017).

[40] NOAA National Centers for Environmental Information. *NCEI/WDS Global Historical Tsunami Database* (National Geophysical Data Center/World Data Service, 2021). 10.7289/V5PN93H7.

[41] Tellman B (2021). Satellite imaging reveals increased proportion of population exposed to floods. Nature.

[42] GDO. GDO Data SPI-3. https://edo.jrc.ec.europa.eu/gdo/php/index.php?id=2112 (2022).

[43] Artés T (2019). A global wildfire dataset for the analysis of fire regimes and fire behaviour. Sci. Data.

[44] Wood HO, Neumann F (1931). Modified mercalli intensity scale of 1931. Bull. Seismol. Soc. Am..

[45] USGS. No Title. https://volcanoes.usgs.gov/vsc/glossary/vei.html (2022).

[46] Pan H, Shi P (2015). World Atlas of Natural Disaster Risk.

[47] Rogers CDW, Kornhuber K, Perkins-Kirkpatrick SE, Loikith PC, Singh D (2022). Sixfold increase in historical northern hemisphere concurrent large heatwaves driven by warming and changing atmospheric circulations. J. Clim..

[48] Giglio, L. MODIS Aqua & Terra 1 km thermal anomalies and fire locations V006 NRT. *Preprint at https://doi.org/10.5067/FIRMS/MODIS/MCD14DL.NRT.006* (2016).

[49] Mukherjee S, Mishra AK, Ashfaq M, Kao SC (2022). Relative effect of anthropogenic warming and natural climate variability to changes in Compound drought and heatwaves. J. Hydrol. (Amst).

[50] Shi Z, Jia G, Zhou Y, Xu X, Jiang Y (2021). Amplified intensity and duration of heatwaves by concurrent droughts in China. Atmos. Res..

[51] Zhang Q (2022). High sensitivity of compound drought and heatwave events to global warming in the future. Earths Future.

[52] BBC News. European weather: Winter heat records smashed all over continent. https://www.bbc.com/news/world-europe-64158283 (2023).

[53] Jones MW (2022). Global and regional trends and drivers of fire under climate change. Rev. Geophys..

[54] Jenkins LJ (2022). The temporal clustering of storm surge, wave height, and high sea level exceedances around the UK coastline. Nat. Hazards.

[55] Lyddon C (2023). Historic spatial patterns of storm-driven compound events in UK estuaries. Estuaries Coasts.

[56] Dent J, Clark C, Holley D (2022). The Brettenham, East Anglia (UK) storm of 25 July 2021: Hydrological response and implications for PMP. Weather.

[57] Baten A, Arcos González P, Delgado RC (2018). OmniScience: A multi-disciplinary journal natural disasters and management systems of Bangladesh from 1972 to 2017: Special focus on flood. Osmj.

[58] Kirschbaum D, Stanley T, Zhou Y (2015). Geomorphology Spatial and temporal analysis of a global landslide catalog. Geomorphology.

[59] Guha-Sapir D, Below R, Hoyois P (2017). EM-DAT The International Disaster Database.

[60] Gill JC, Malamud BD (2014). Reviewing and visualizing the interactions of natural hazards. Rev. Geophys..

[61] Franklin JL (2006). Atlantic hurricane season of 2004. Mon. Weather Rev..

[62] Villarini G, Smith JA, Baeck ML, Marchok T, Vecchi GA (2011). Characterization of rainfall distribution and flooding associated with U.S. landfalling tropical cyclones: Analyses of Hurricanes Frances, Ivan, and Jeanne (2004). J. Geophys. Res. Atmos..

[63] Claassen, J. N. *et al.* MYRIAD – Hazard Event Sets (MYRIAD-HES). 10.5281/ZENODO.8269680 (2023).

