# Supplementary material for: A new method to compile global multi-hazard event sets
Source: Sci Rep. 2023 Aug 23;13:13808. doi: 10.1038/s41598-023-40400-5 (PMC10447514; doi:10.1038/s41598-023-40400-5)
Supplement: Supplementary file 1 — Supplementary Information 1. [file 41598_2023_40400_MOESM1_ESM.docx]

Supplementary Materials

A New Method to Compile Global Multi-Hazard Event Sets

Judith N. Claassen^1*^, Philip J. Ward^1,2^, James Daniell^3,4^, Elco E. Koks^1^, Timothy Tiggeloven^1^, and Marleen C. de Ruiter^1^

^1^ Institute for Environmental Studies, Vrije Universiteit Amsterdam, Amsterdam, The Netherlands ([j.n.claassen@vu.nl](mailto:j.n.claassen@vu.nl))

^2^ Deltares, Delft, The Netherlands

^3^ Risklayer GmbH, Karlsruhe, Germany

^4^ CEDIM, Karlsruhe Institute of Technology, Karlsruhe, Germany

Table 1: Percentage of land that has each hazard pair as the most frequent (ocean not included)

| **combinations** | **percentage** |
| --- | --- |
| NaN | 42.3128 |
| dr & hw | 19.33549 |
| hw & ew | 16.29381 |
| tc & ew | 2.440599 |
| dr & cw | 2.322209 |
| ew & cw | 2.2161 |
| dr & ew | 2.159385 |
| hw & wf | 2.06924 |
| fl & ew | 2.01739 |
| fl & hw | 1.817634 |
| fl & fl | 1.110165 |
| fl & dr | 1.093246 |
| dr & wf | 0.813169 |
| ew & wf | 0.760683 |
| tc & hw | 0.634562 |
| dr & tc | 0.430303 |
| fl & tc | 0.348709 |
| dr & ts | 0.34457 |
| eq & ts | 0.301226 |
| tc & tc | 0.282033 |
| fl & cw | 0.255198 |
| eq & dr | 0.121618 |
| eq & eq | 0.080548 |
| fl & wf | 0.067631 |
| ls & ls | 0.056306 |
| cw & wf | 0.037977 |
| ts & cw | 0.033975 |
| fl & ls | 0.02688 |
| vo & ew | 0.026334 |
| vo & hw | 0.025424 |
| eq & cw | 0.02497 |
| eq & hw | 0.022786 |
| eq & ls | 0.021831 |
| dr & ls | 0.018193 |
| ts & ew | 0.014964 |
| tc & ls | 0.011098 |
| ls & ew | 0.009779 |
| dr & vo | 0.007323 |
| ts & hw | 0.005276 |
| ls & hw | 0.005276 |
| eq & fl | 0.003593 |
| eq & tc | 0.003138 |
| tc & wf | 0.003093 |
| vo & tc | 0.002001 |
| fl & vo | 0.001819 |
| ts & ts | 0.001592 |
| ts & tc | 0.00141 |
| ts & wf | 0.001183 |
| eq & wf | 0.00091 |
| eq & vo | 0.000864 |
| ts & vo | 0.000773 |
| fl & ts | 0.000728 |
| vo & wf | 0.000682 |
| eq & ew | 0.000682 |
| ts & ls | 0.000637 |
| ls & cw | 0.000136 |
| vo & cw | 4.55E-05 |
| ls & wf | 0 |
| hw & cw | 0 |
| cw & cw | 0 |
| hw & hw | 0 |
| ew & ew | 0 |
| tc & cw | 0 |
| vo & ls | 0 |
| vo & vo | 0 |
| dr & dr | 0 |
| wf & wf | 0 |


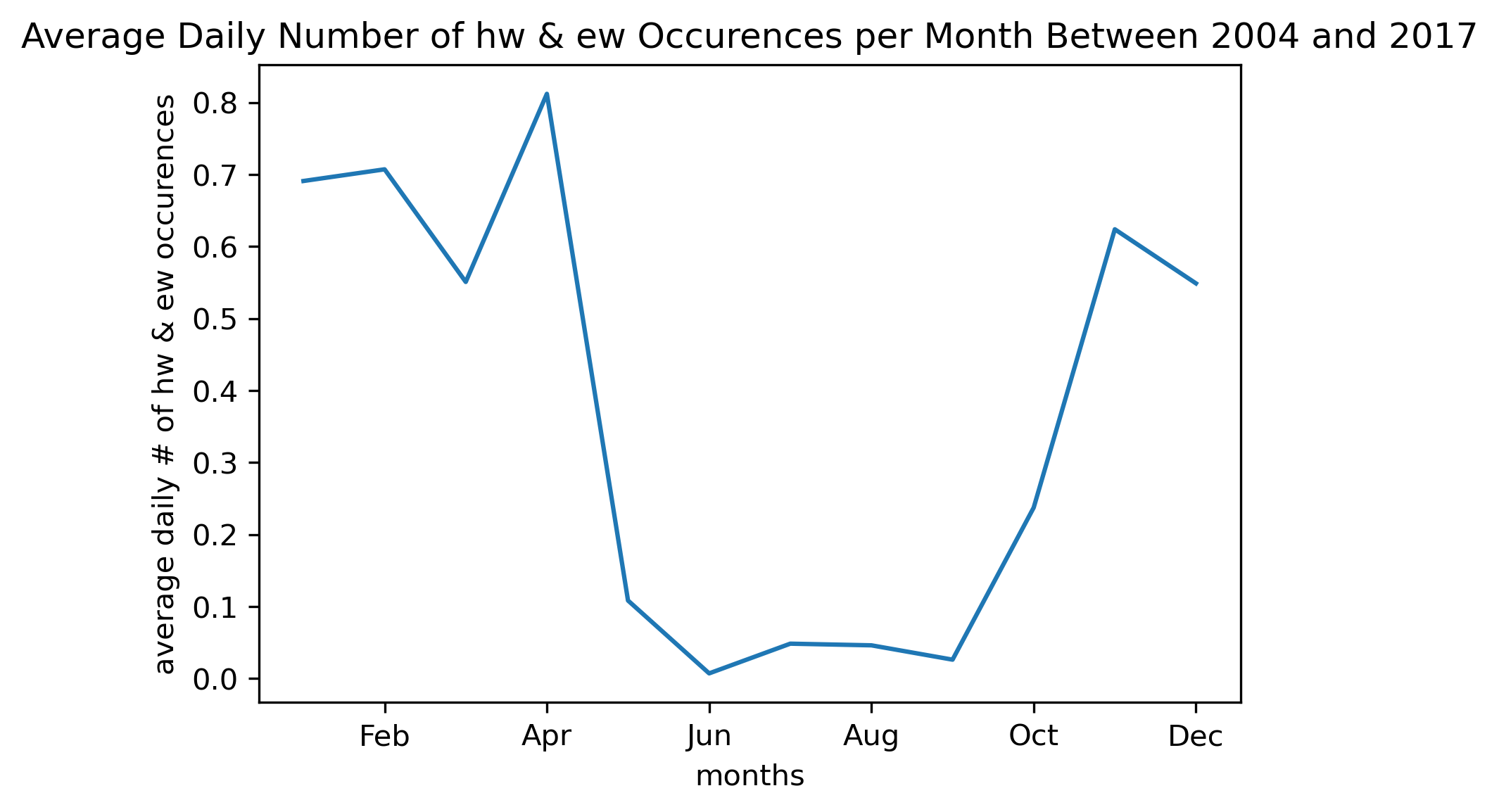


Figure 1: Seasonality of Heatwave and Extreme wind pair in Europe

Table 2: Number of events, average duration and median area of each single hazard between 2004 and 2017

| **Hazard type** | **Number of events** | **Average days** | **Median area [km^2^]** |
| --- | --- | --- | --- |
| Earthquake | 2289 | 0 | 1507.101 |
| Volcanic eruption | 496 | 186 | 431.099 |
| Landslide | 4355 | 0 | 351.9473 |
| Tropical Cyclone | 504 | 3 | 1050222 |
| Cold-wave | 26636 | 2 | 909.4891 |
| Heatwave | 161681 | 2 | 890.4209 |
| Extreme wind | 40121 | 0 | 6793.175 |
| Tsunami | 210 | 0 | 92786.08 |
| Flood | 707 | 19 | 6357.332 |
| Drought | 7439 | 61 | 22629.67 |
| Wildfire | 1344115 | 9 | 10.04941 |


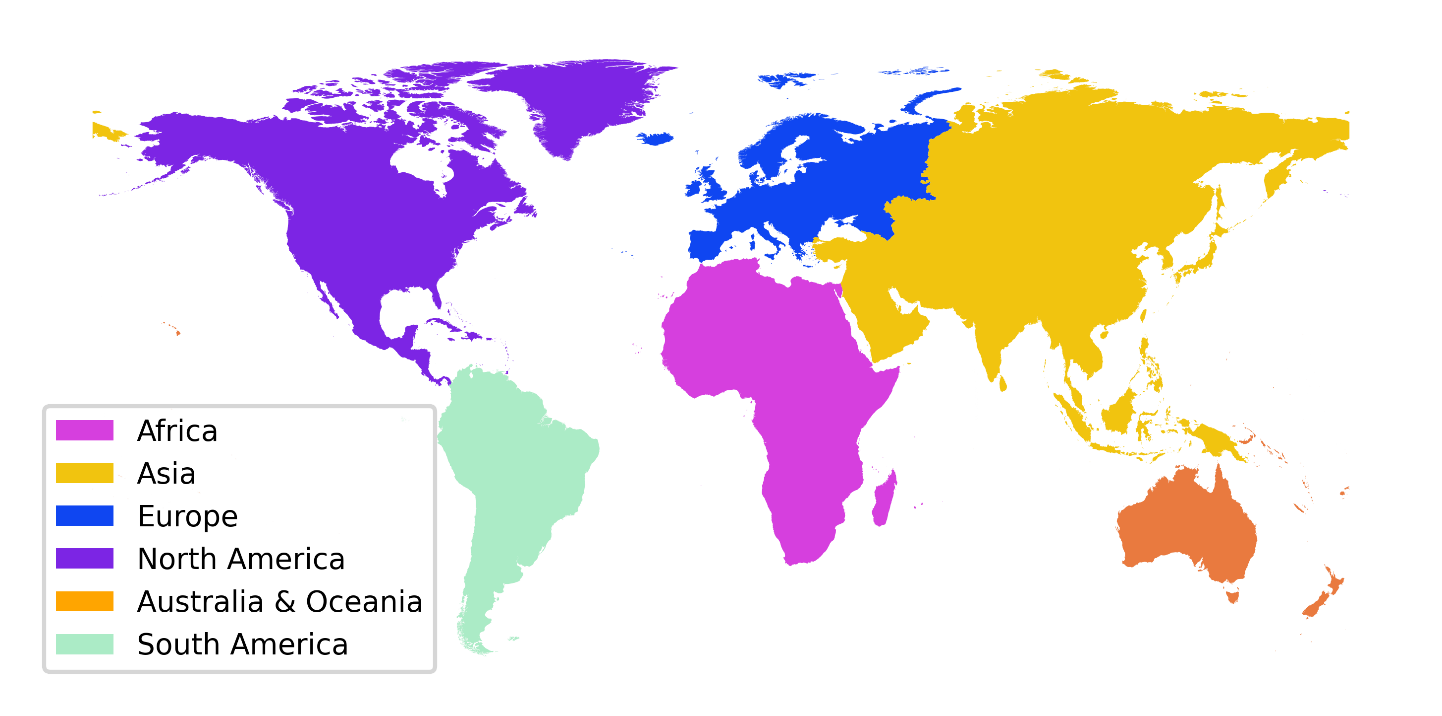


Figure 2: The different continents used for Fig. 6


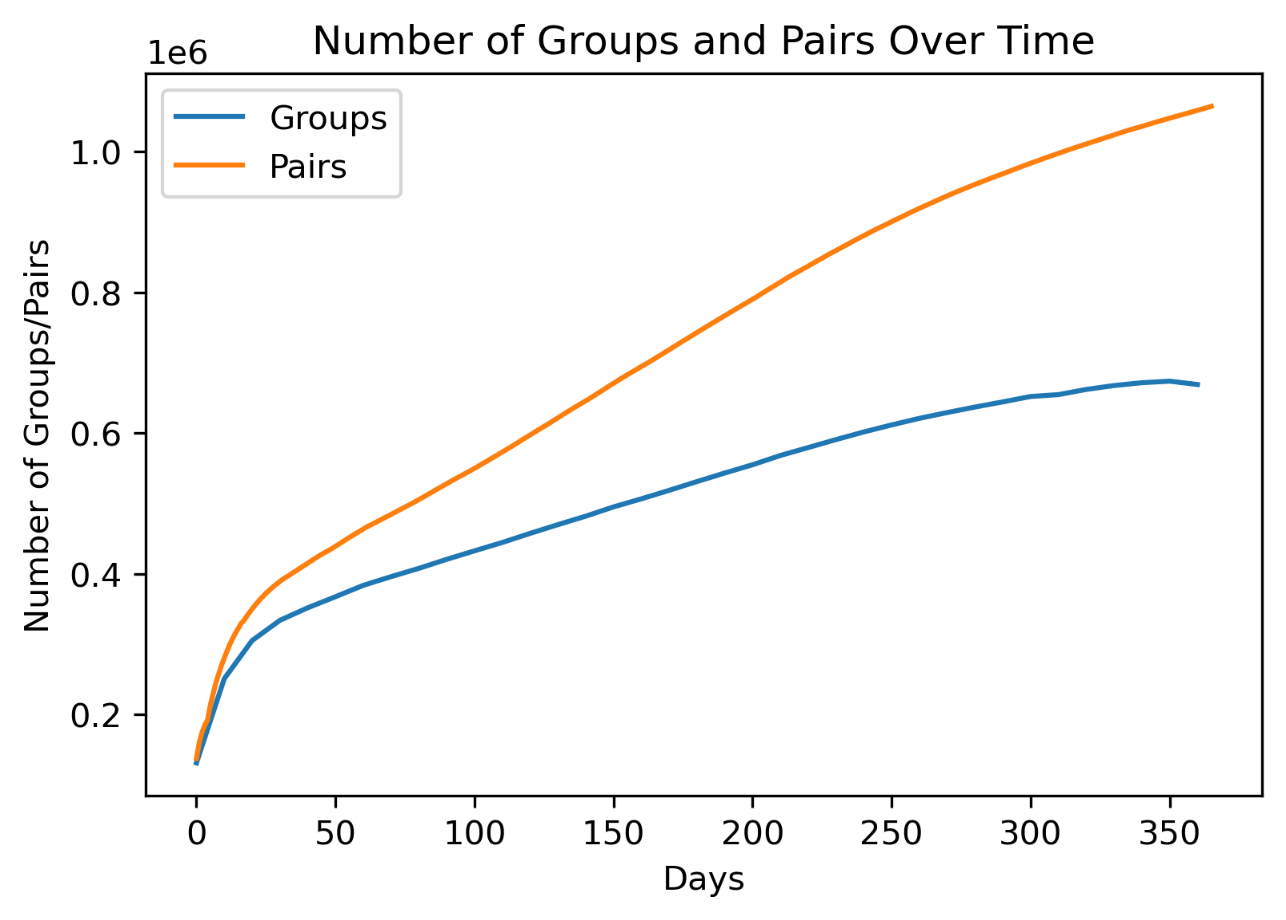


Figure 3: The number of hazard pairs and groups with varying time-lags
